# Feasibility of reduced PTV margins in magnetic resonance image-guided online adaptive radiotherapy for bladder cancer: a personalised approach

**DOI:** 10.1016/j.ctro.2026.101321

**Published:** 2026-10-10

**Authors:** Bethany Williams, Saleh Sandoughdaran, Joan Chick, Alex Dunlop, Trina Herbert, Robert Huddart, Adam Mitchell, Simeon Nill, Shaista Hafeez, Helen A McNair

**Affiliations:** a The Royal Marsden NHS Foundation Trust, London, United Kingdom; b The Institute of Cancer Research/The Royal Marsden NHS Foundation Trust, London, United Kingdom; c Joint Department of Physics at the Royal Marsden and The Institute of Cancer Research, United Kingdom

**Keywords:** Bladder cancer, Online adaptive radiotherapy, Margin reduction, MRI-guided radiotherapy, Personalisation

## Abstract

**Introduction:**

Bladder MRI-guided online adaptive radiotherapy (MRIgART) has enabled a reduction in planning target volume (PTV) margins, reducing normal tissue irradiation. Yet, further margin reduction is limited due to workflow duration and intra-fractional bladder variation. This study reports the feasibility of a 5 mm isotropic PTV in whole bladder MRIgART, 55 Gy in 20 fractions daily, and 36 Gy in 6 fractions weekly, with a personalised approach to margin expansion.

**Methods:**

Patients voided their bladders prior to treatment. Following setup, a daily MRI (MR_DAILY_) was acquired, and an adaptive workflow was performed. An MRI was acquired during delivery to coincide with treatment completion (MR_POST_). Time from MR_DAILY_ to MR_POST_ was recorded. Offline, target coverage of the clinical target volume (CTV) was visually monitored by comparing MR_DAILY_ and MR_POST_ images. Images were reviewed by the radiation oncologist (RO) if the CTV was outside of the PTV on the MR_POST_. Under RO discretion, margins were personalised to the minimum margin that would have encompassed the CTV and applied online for subsequent fractions. In retrospective analysis, MR_POST_ CTVs were recontoured, and the adaptive plan recalculated. CTV coverage was deemed acceptable if V95% ≥ 95%. If this objective was not met, the gross tumour volume (GTV) was delineated and the dose reported; coverage was deemed acceptable if GTV V95% ≥ 95%.

**Results:**

A total of 138 fractions from ten patients were analysed. Median (interquartile range) workflow time was 25.6 (20.8–30.7) minutes. PTV margins were personalised for 31 fractions in three patients. 119 MR_POST_ images were available for analysis. CTV coverage was achieved in 94% (112/119) of fractions. Where the CTV mandatory objectives were missed (6%; 7/119 fractions), GTV coverage was acceptable in 6 of the 7 fractions.

**Conclusion:**

Whole bladder MRIgART with 5 mm isotropic PTV margins with a personalised approach to margin expansion is feasible in achieving acceptable CTV coverage.

## Highlights

• 5 mm isotropic PTV margins are feasible in MRI-guided online adaptive whole bladder radiotherapy.
• Margin personalisation accounts for patient-specific intra-fractional variability.
• Online margin personalisation offers an alternative to population-based margins.

## 1 Introduction

Bladder radiotherapy offers an organ-preserving treatment option for those with bladder cancer [1], [2]. By convention, the whole bladder is irradiated. However, the bladder presents a complex target for radiotherapy, posing inter- and intra-fractional deformations due to urinary filling and displacement by adjacent organs [3], [4], [5]. This variability is influenced by patient-specific factors, including hydration, extent of disease, urinary function and individual anatomy [3], [4], [5], [6], [7]. Historically, this uncertainty has been accounted for with extensive population-based planning target volume (PTV) margins, at the expense of normal tissue irradiation [3], [4], [5].

One adaptive radiotherapy solution utilising cone-beam computed tomography (CBCT) is the ‘plan of the day’ (POTD) approach, which employs a plan library with varying PTV margins selected based on daily anatomy [1], [2], [8]. POTD has demonstrated PTV reductions of 25–40% whilst improving target coverage [8] and has enabled tumour-focused dose-escalated adaptive radiotherapy (DART) without increased toxicity compared to whole bladder radiotherapy [9].

Building on this, online adaptive radiotherapy (oART) enables the redefinition of target volumes and the optimisation of treatment plans at each fraction, aiming to further enhance target accuracy and toxicity profiles [4], [10], [11]. However, the extended workflow duration of oART is a limiting factor in PTV margin reduction in bladder radiotherapy, primarily due to intra-fractional urinary filling. Consequently, large PTV margins of up to 15 mm have still been applied [10], [12], mitigating the benefit of daily redefined target volumes and failing to account for patient-specific factors.

A preliminary retrospective margin reduction analysis indicated that 5 mm isotropic PTV margins could achieve clinically acceptable target coverage in most patients when using MRIgART [13]. Following implementation, this dosimetric analysis reports the feasibility of 5 mm isotropic PTV margins for whole bladder MRIgART on the Elekta Unity MR Linac (MRL) (Elekta AB, Stockholm, Sweden), for patients prescribed 55 Gy in 20 fractions (fr), daily, and 36 Gy in 6 fr, weekly, with a personalised approach to margin expansion to counter patient-specific variability.

## 2 Methods

### 2.1 Online workflow

Patient selection comprised consecutive patients receiving whole bladder radiotherapy (only) on the MRL, as per standard clinical indication. All patients were asked not to drink fluids for 2 h and to void their bladder immediately prior to each fraction. Following patient set-up, a 1.1 min T2 weighted (T2w) MRI sequence (MR_DAILY_) [14] was acquired and registered to a reference image. For fraction one, the reference image was the initial planning CT. Fig. 1 presents the adapt-to-shape (ATS) workflow previously described [10], using the Monaco treatment planning system (v.6.2.1, Elekta AB, Stockholm, Sweden). Structures were propagated from the reference image and edited; the adaptive plan was optimised and approved on fraction one by a radiation oncologist (RO) (Fig. 1). This was used as the reference plan for remaining fractions, with contouring by therapeutic radiographers (RTT) and plan approval by a delegated operator (medical physicist) [15].

**Fig. 1:**
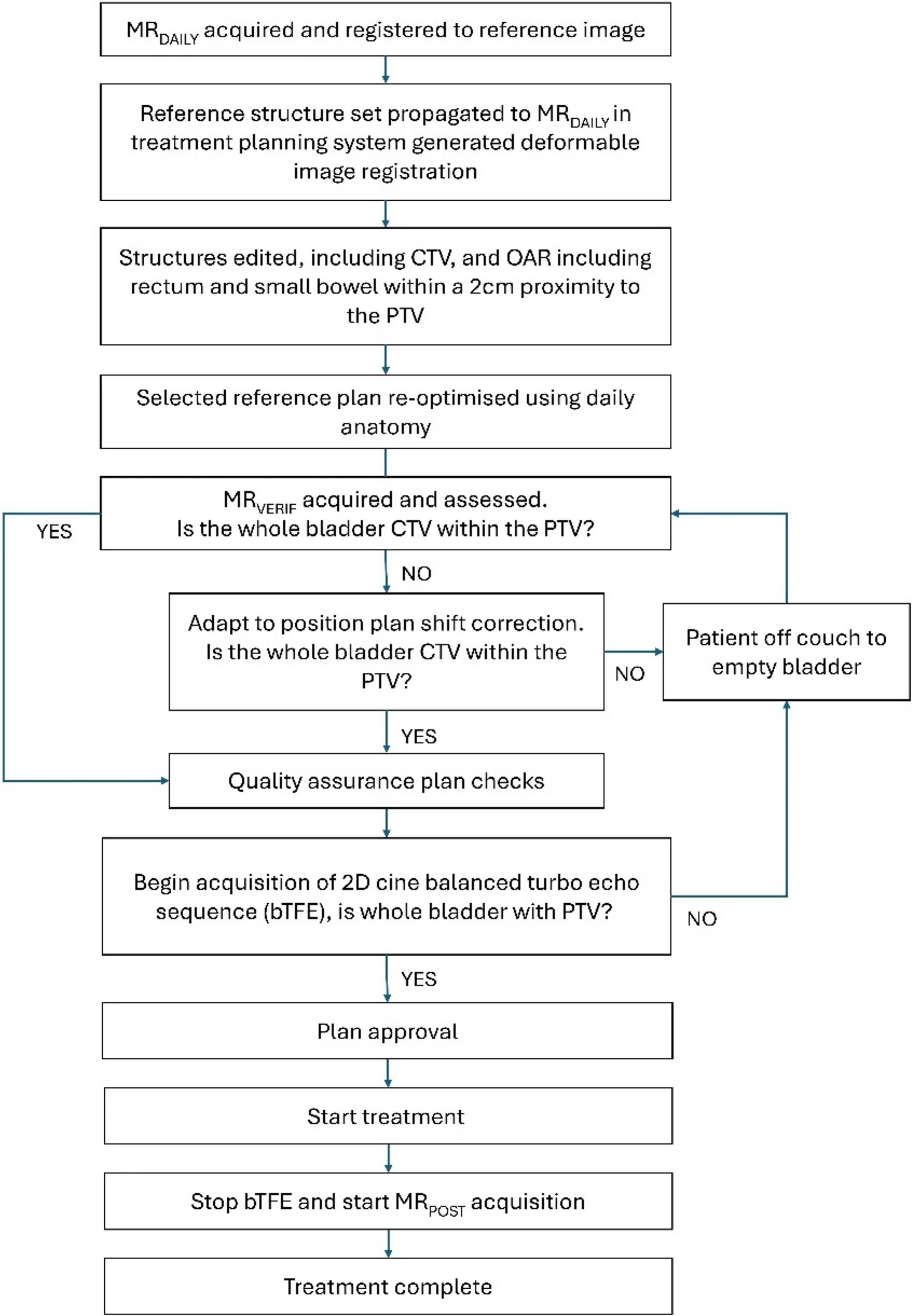
Flowchart of online ATS workflow.

During plan optimisation, an MR verification (MR_VERIF_) image was acquired and compared to MR_DAILY_. In cases of intra-fractional change, we performed a correction via the previously described adapt-to-position (ATP) workflow [10]. Where the visible target was outside the PTV after ATP image registration, the patient was removed from the couch, asked to empty their bladder, and the ATP workflow was resumed (Fig. 1)_._ The number of ATPs and the frequency with which patients were removed from the couch were recorded.

A 2D cine balanced turbo fast echo sequence (bTFE) was acquired to review target coverage prior to and during initial treatment delivery. During treatment, the bTFE was stopped at approximately 50% of beam delivery to acquire a 2-min T2w sequence (MR_POST_) to coincide with treatment completion. The MR_POST_ provided a 3D image data set serving as a surrogate for anatomy during treatment, enabling dosimetric analysis and estimation of the dose delivered with intra-fractional change. The time from MR_DAILY_ to MR_POST_ was recorded.

### 2.2 Margin personalisation

All patients started treatment with 5 mm isotropic PTV margins. Visual inspection of the CTV coverage was performed by comparing MR_DAILY_ with MR_POST_ during an offline weekly review, unless concerns were raised during treatment, in which case assessment occurred post-treatment. Patient images were escalated by the RTT for RO review prior to the next fraction if: the bladder (CTV) filled outside of the PTV on MR_POST_ or consecutive ATP workflows were required. The RO assessed the images for inadequate CTV coverage and determined whether intra-fractional change would be clinically significant, considering patient-specific factors such as bladder tumour location. At the RO's discretion, PTV margins were amended at the subsequent online fraction to the minimum margin that would have encompassed the visible CTV. The adaptive plan with margin personalisation was used as the reference plan for all subsequent fractions.

### 2.3 Retrospective offline dosimetric analysis

All patients consented to the use of their images for research under PERMIT (NCT03727698) and MOMENTUM (NCT04075305). An offline retrospective analysis was performed when each patient had completed their treatment course. For each fraction, the MR_DAILY_ structures were propagated to the corresponding MR_POST_ in Monaco. The CTV was re-edited by one RTT, the volume difference between MR_DAILY_ and MR_POST_ was recorded and the online adaptive treatment plan was recalculated on the MR_POST_. Target coverage was deemed acceptable if 95% of CTV received ≥95% of the prescribed dose (V95% ≥ 95%). If this objective was not met, the bladder tumour (GTV) was contoured by an RO on the MR_POST,_ and the dose was reported for purposes of retrospective analysis only. Coverage was deemed acceptable if GTV V95% ≥ 95%. In patients with multi-focal disease within the bladder, target coverage was assumed unacceptable if the mandatory CTV objectives were missed.

## 3 Results

Ten patients with muscle-invasive bladder cancer suitable for whole bladder radiotherapy were recruited for analysis. Patient characteristics are summarised in Table 1. A total of 138 fractions were analysed; one fraction of one patient receiving 6fr, and five fractions from two patients receiving 20fr were treated on standard linear accelerators due to the MRL being unavailable.

**Table 1:** Patient characteristics.

Patient	Age	Gender	TNM stage	Location of bladder tumour	Fractions (n)			
					Planned on MRL	Treated on MRL	Interrupted due to bladder emptying	ATP required
1	61	Male	T2N0M0	Right vesicoureteral junction (unifocal)	20	20	1	3
2	82	Male	T2N0M0	Right lateral bladder wall (unifocal)	20	20	5	6
3	85	Male	T2N0M0	Right lateral bladder wall (unifocal)	20	18	5	5
4	80	Male	T2N0M0	Bladder dome (unifocal)	20	20	1	18
5	79	Female	T2N0M0	Right bladder dome (unifocal)	20	17	2	5
6	79	Male	T1b/T2N0M0	Right lateral bladder wall (multifocal)	20	20	3	3
7	84	Male	T1/T2N0M0	Right lateral bladder wall (unifocal)	6	6	0	1
8	91	Male	T1b/T2N0M0	Right lateral bladder wall and neck (unifocal)	6	6	2	2
9	88	Female	T2/T3bN0M0	Left lateral and anterior bladder wall (multifocal)	6	6	0	0
10	87	Male	T2/T3N0M0	Left lateral bladder wall (unifocal)	6	5	0	1

The median (IQR) filling rate from MR_DAILY_ to MR_POST_ was 0.6 (0.2–0.9) cc/min and 0.2 (0.1–0.5) cc/min, for the 20fr and 6fr cohort, respectively. ATP frequency median (range) was 5 (3–18) and 1 (0–2) per treatment course for the 20fr cohort and 6fr cohort. Patient four exhibited disproportionate number of ATPs (18) due to atypical bowel motion (Fig. 2). The median (range) number of times patients were required to empty their bladder mid-workflow was 1.5 (0–5) for all patients, 3 (1–5) and 0 (0–2) for 20fr and 6fr, respectively. Median (interquartile range, IQR) workflow time (MR_DAILY_ to MR_POST_) was recorded for 122 fractions, including fractions in which the workflow was interrupted for bladder emptying, was 26.6 (21.0–34.1) minutes (Fig. 3). For those fractions where no interruption was incurred (106/122 timed fractions), the median workflow time was 25.6 (20.8–30.7) minutes (Fig. 3b).

**Fig. 2:**
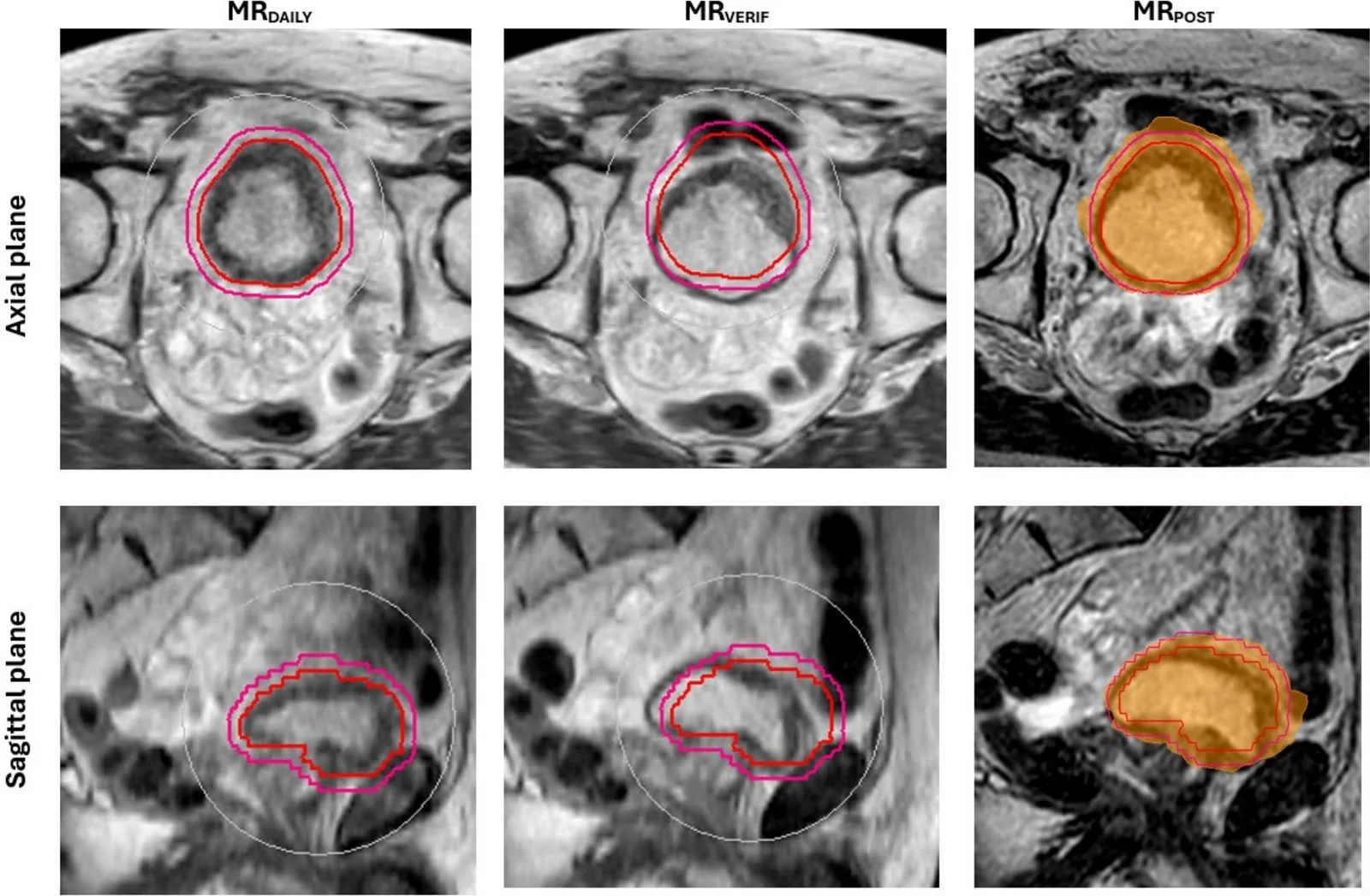
Patient 4, fraction 6, example of atypical intra-fractional variation in the position of small bowel anteriorly causing displacement of the bladder position (PTV pink, CTV red) from MR_DAILY_ (left column), to MR_VERIF_ prior to ATP correction (middle column), to MR_POST_ acquired during treatment following ATP correction with 95% isodose in orange (right column). (For interpretation of the references to colour in this figure legend, the reader is referred to the web version of this article.)

**Fig. 3:**
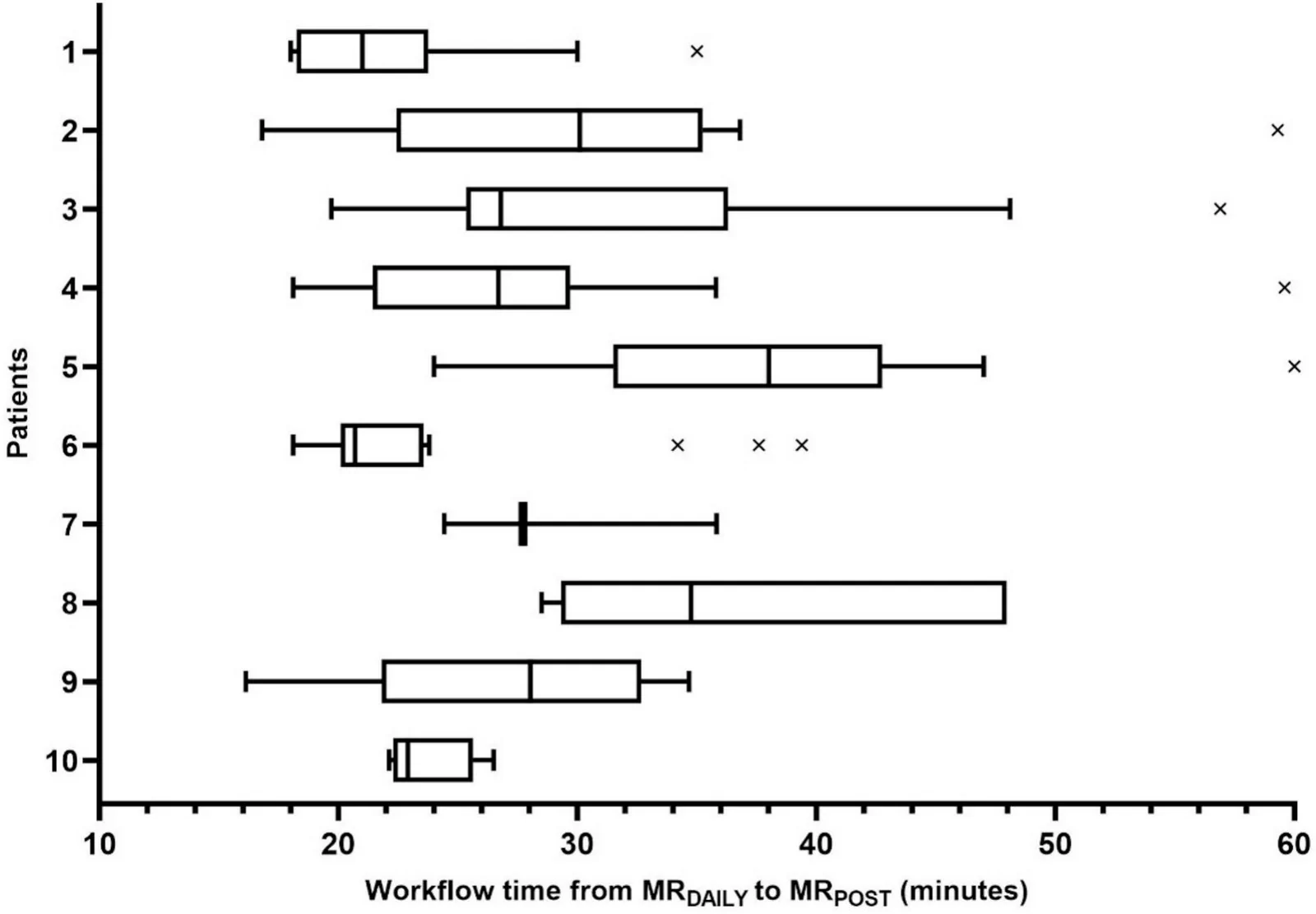

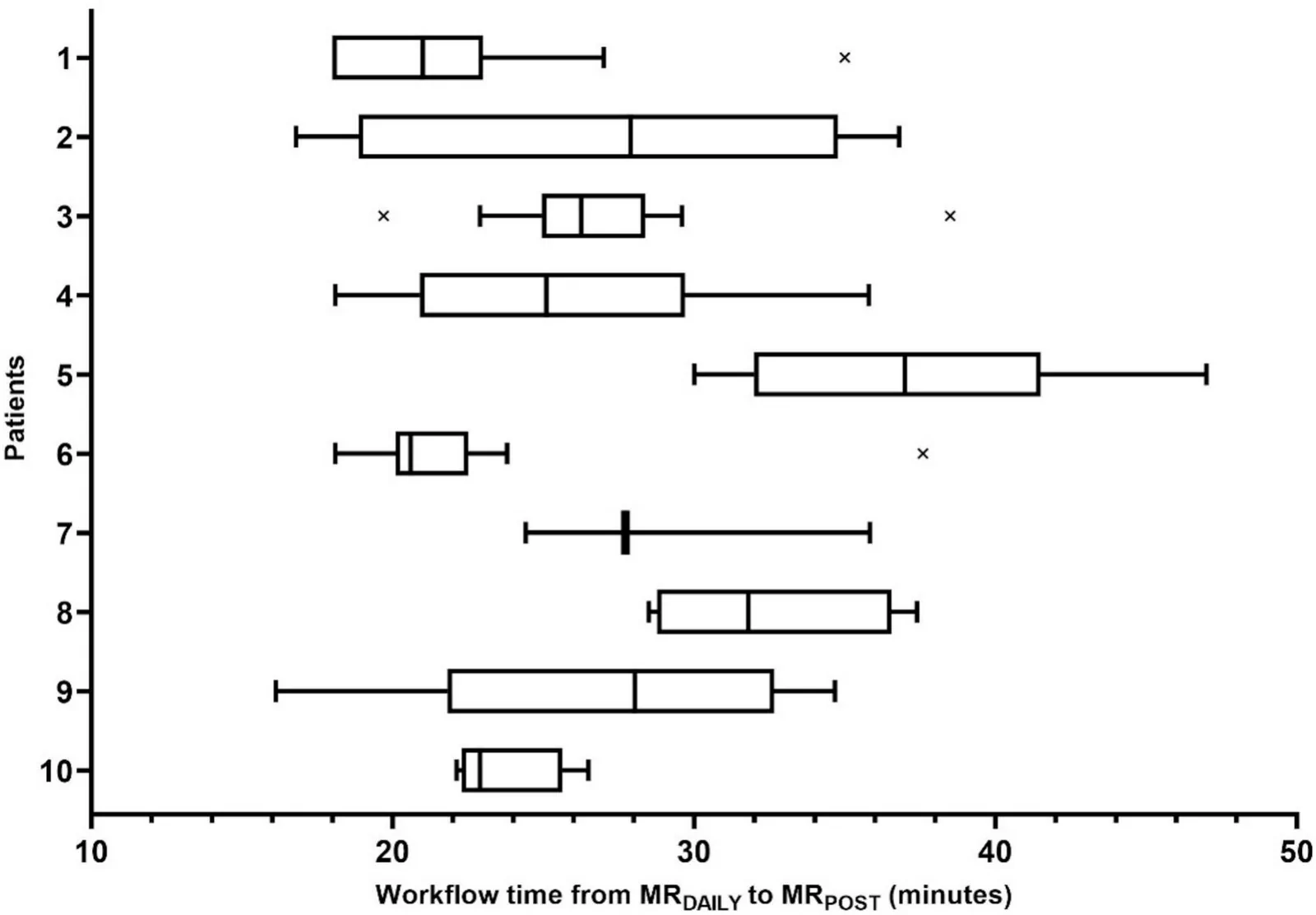
a. Box plot of median IQR (25th–75th percentile) workflow time from MR_DAILY_ to MR_POST_ in minutes, including fractions where the workflow was interrupted for bladder emptying. b. Box plot of median IQR (25th–75th percentile) workflow time from MR_DAILY_ to MR_POST_ in minutes, excluding fractions where the workflow was interrupted for bladder emptying.

Of the 138 fractions, 119 MR_POST_ images were available for retrospective analysis, 101 and 18 for 20fr and 6fr cohorts, respectively. For the remaining fractions, MR_POST_ images were either not acquired or unusable. Overall, CTV coverage was acceptable in 94% (112/119) of fractions (Fig. 4), 93% (94/101) of the 20fr cohort fractions, and 100% (18/18) of the 6fr cohort fractions. The CTV mandatory objective was missed in 7/101 fractions in the 20fr cohort only: three times for patient 2 (twice post-margin increase), twice for patient 4, and once for patients 5 and 6 (Table 2). GTV coverage was acceptable in 6 of these 7 fractions. It was assumed unacceptable without analysis for patient 6 because the disease was multifocal. The incidence in patient 6 occurred after hydration during concurrent chemotherapy administration. For the other instances where the mandatory CTV constraints were not met, no identifiable cause was found.

**Fig. 4:**
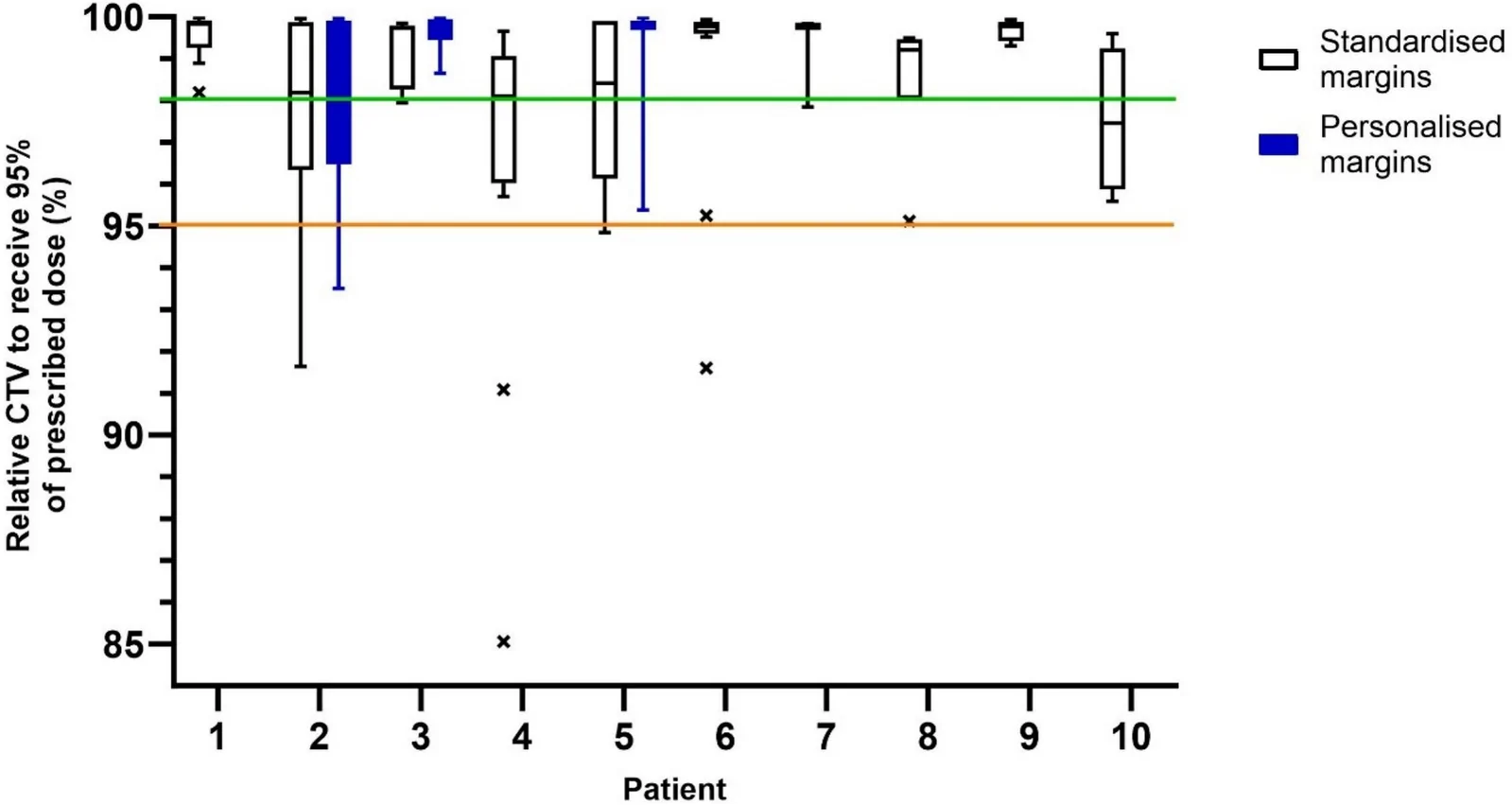
Box plot of the relative CTV to receive 95% of the prescribed dose of patients with optimal (green line) and mandatory constraints (orange line). Data is shown as median IQR (25th–75th percentile). (For interpretation of the references to colour in this figure legend, the reader is referred to the web version of this article.)

**Table 2:** Number of fractions per patient where the mandatory CTV constraint was not met, with the standardised PTV margin (5 mm isotropic) and personalised PTV margins.

Patient	Standardised PTV (n)	Personalised PTV (n)
1	0	N/A
2	1	2
3	0	0
4	2	N/A
5	1	0
6	1	N/A
7	0	N/A
8	0	N/A
9	0	N/A
10	0	N/A

Of the 91 fractions available prior to margin personalisation, 5 mm PTV margins achieved acceptable CTV coverage in 95% (86/91) of fractions, 93% (68/73) and 100% (18/18) of the 20fr and 6fr cohort fractions, respectively. PTV margins were personalised for three patients (Fig. 4), on fractions 9–10. Two patients (patients 2 and 3) increased to 8 mm anisotropic superiorly due to intra-fractional filling. One patient (five) increased to 8 mm in all directions except 10 mm posteriorly, due to variable intra-fractional deformation and filling due to an implant. For these patients, 20/28 fractions were available for analysis before the personalised margins were implemented, 90% (18/20) achieved acceptable CTV coverage. Versus after margin increase, 28/31 fractions were available, 93% (26/28) achieved acceptable coverage.

## 4 Discussion

To our knowledge, this study is the first to report the feasibility of reducing PTV margins in MRIgART for bladder cancer through a dosimetric analysis. Results demonstrate that a 5 mm isotropic PTV achieves clinically acceptable target coverage in 95% of fractions (Fig. 4). This is consistent with our preliminary margin reduction analysis, which reported that CTV coverage could be maintained in 96% of fractions across 7 patients [13].

Both MRL analyses investigated the use of the smallest clinically implemented PTV margin in POTD adaptive treatments delivered on standard linear accelerators within the same department. Compared with MRIgART, a typical POTD workflow is likely to have a shorter treatment time and therefore be less susceptible to intra-fractional anatomical changes. Nevertheless, this study's results suggest that a 5 mm isotropic PTV is feasible for MRIgART's intrinsically longer workflows. These findings suggest that PTV margin reduction may be achievable in MRIgART, potentially increasing normal tissue sparing while maximising the clinical benefits of its improved inter- and intra-fractional motion mitigation compared with POTD approaches [4], [10]. Previous studies have shown the anticipated benefit to normal tissue irradiation when PTV margin reduction is applied in bladder radiotherapy [1], [2], [5], [8]. Notably, our study is a dosimetric analysis of target coverage only; investigation quantifying reduction in normal tissue irradiation should be considered in future analysis.

Application of a 7 mm isotropic PTV has been reported in bladder CT-guided oART (CTgART) [16], [17]. Across 10 patients CTV median V100% was reported as 96.1% and 92.9% on verification imaging for high and low dose, respectively, in a median workflow of 19.7 min [17]. While verification imaging ensured treatment accuracy prior to beam on, we analysed during-treatment images, so estimating dose delivered when intra-fractional change occurs. Other than a slightly smaller PTV, this explains the lower percentage of fractions meeting CTV objectives in our study. The analysis reported displacements in fractions over 5 mm in cranial-caudal and anterior-posterior directions, averaged at 4–5% [17], suggesting that PTV margins of less than 7 mm could be feasible while maintaining adaptive plan quality.

Numerous studies have attempted to quantify inter- and intra-fractional bladder deformation, applying population-based margins to account for organ motion. Yet margin development, such as the Van Herk recipe [18], is designed for solid targets and fails to account for patient-specific factors [12]. Our study demonstrates an alternative to population-based margins in bladder oART. The protocol utilised a personalised approach to margin expansion based on contemporaneous image review, which aimed to monitor variability in patient-specific bladder deformation while leveraging the opportunity in oART to increase margins online, without the resource burden of offline replanning.

Margin personalisation was applied in 30% (3/10) of patients. For these three patients, the percentage of fractions achieving acceptable CTV coverage was increased from 90% to 93%. Before personalisation, CTV coverage was satisfactory for most fractions. Objectively, personalisation had little impact on improving target coverage; yet, subjectively, target coverage was more secure and reduced the need for ATP and additional workflow interruptions for bladder emptying. Meanwhile, preventing patients with minimal intra-fractional change being subject to unnecessary irradiation of normal tissue. This is demonstrated by the remaining 7 patients, in whom 96% (68/71) of the fractions met acceptable CTV coverage with a 5 mm PTV (Fig. 4).

It is acknowledged that, firstly, for patients who require increased margins, CTV coverage may be compromised prior to personalisation, yet verification and intra-fractional imaging ensure target coverage is monitored. Secondly, that margin personalisation alone may not fully compensate for intra-fractional change in all fractions yet will reduce chance of CTV underdosage. The preliminary results of this small cohort study demonstrate the feasibility of implementing selective margin adaptation, but further investigation is required to prove margin personalisation improves target coverage.

In whole bladder radiotherapy CTV coverage is commonly chosen as the clinically relevant endpoint. However, we have chosen to also illustrate coverage specifically to the GTV in fractions where CTV V95 > 95% was not achieved, as target compromise to this region carries the highest risk of relapse. Relapses after bladder radiotherapy most commonly occur at the original tumour site [19], [20]. Bladder tumour-focused radiotherapy shows that a low dose to the uninvolved bladder in unifocal muscle-invasive disease does not compromise local disease control [9], [21], [22]. We therefore chose dose assessment to GTV itself as a much more rigorous assessment of clinical practice and potential impact, particularly where CTV coverage appeared sub-optimal.

7/119 evaluated fractions failed to meet the CTV mandatory constraint (Table 2). GTV coverage was acceptable in 6 of these 7 fractions. Given the unpredictable nature of inter- and intra-fractional bladder deformation, isolated fractions of target underdosage are an inherent limitation of bladder radiotherapy regardless of margin size and, to some degree, is implicitly tolerated in clinical practice. We assume that any compromise demonstrated in this study is not clinically significant, particularly when considering cumulative dose. Nonetheless, further evaluation, including assessment of clinical outcomes, is required, as the impact otherwise remains uncertain.

Future work should evaluate accumulated CTV dose over the entire treatment course, as per-fraction target coverage in oART does not necessarily reflect the cumulative dose delivered. While isolated reductions in CTV coverage may have limited clinical impact, repeated underdosage in the same region could result in clinically significant cold spots. Conversely, if underdosage occurs in different regions between fractions, the cumulative dose distribution may remain acceptable. Consequently, dose accumulation analysis may provide a more clinically meaningful assessment of the adequacy and safety of the reduced-margin strategy than per-fraction dosimetric evaluation alone.

Bladder deformation most commonly occurs in the superior and anterior regions [3], [6], [7], [16], [17]. This is reflected in two patients who had margin personalisation, in which superior anisotropic margins were applied due to intra-fractional filling. While empty-bladder protocols have been shown to reduce intra-fractional filling [3], [17], this depends on patient-specific factors such as hydration, disease extent, and urinary function [3], [4], [5], [6], [7]. This is demonstrated by a lower filling rate in the 6fr cohort, who are typically of poorer performance status, compared to the 20fr cohort (0.2 cc/min versus 0.6 cc/min) an observation seen in previous studies [8].

Daily bladder volume has also been reported to decrease throughout a treatment course of bladder radiotherapy [12], potentially related to adverse effects [6]. However, like many others, we found no consistent trend [3]. Nonetheless, this highlights the benefit of flexible margin protocols that enable online margin personalisation during treatment. While this study reported only increased margins, margins could decrease with a decreasing filling rate or catheterisation.

Another example of patient-specific variability is patient 5, who had anisotropic increases to 8 mm in all directions except for 10 mm posteriorly. This was due to variable intra-fractional deformation from an implant, which also caused image artefact, increasing contouring time (Fig. 1). Retrospective analysis identified one fraction missed mandatory CTV constraints prior to margin personalisation only (Table 2). Patient 4 exhibited atypical anterior bowel motion that displaced the bladder posteriorly intra-fractionally; hence, multiple ATP corrections were required. In this scenario, the margin increase was not applied, as visual assessments deemed ATP corrections appropriate to compensate for intra-fractional changes (Fig. 2), despite an inherent increase in workflow time. Subsequent dosimetric analysis of patient 4 identified two fractions in which the mandatory CTV constraints were not met. However, given the sporadic nature of bowel motion, it is unclear whether increasing the PTV margin would have achieved target coverage without exceeding small-bowel constraints in these instances.

While dosimetric analysis showed that margin personalisation was sufficient to moderately improve target coverage for the remaining fractions, it was based on visual assessment and clinical judgement by ROs and therefore subject to inter-observer variability. Although the results demonstrate this was an appropriate decision for patients 2 and 5, for patient 3, CTV coverage was acceptable before the margin increase and may have been deemed unnecessary following dosimetric insight (Fig. 4). This patient had extensive disease, which may have affected inter- and intra-fractional variability in filling [6]. Alternatively, in two fractions post margin increase, patient 2 still failed to meet CTV objectives despite margin personalisation (Table 2). This highlights that visual assessment alone is subjective and could result in either unnecessary or inadequate margin increase. No retrospective analysis was conducted to evaluate what target coverage would have been if margins had not been increased.

A CTgART study developed patient-specific adaptive margin recipe equations for bladder radiotherapy based on CBCTs from the first 5 treatment fractions. Across 16 patients, the approach resulted in an average PTV margin of 6 mm, optimising normal tissue sparing while maintaining adequate PTV coverage [12]. Margin recipes could be a more accurate way to determine margin personalisation. However, the resource burden of implementing detailed offline review into radiotherapy services must be considered, particularly as oART is already deemed more resource-intensive than non-adaptive radiotherapy. A previous publication suggested that using POTD PTV margins that cover 95% or 90% of CTV could balance target coverage, adverse events, and resource use [23]. Long-term clinical outcomes are awaited; there are few publications reporting toxicity outcomes in reduced-margin bladder MRIgART. Further research is required to establish the clinical impact and potential patient benefits, which may then justify increased implementation of margin personalisation. Advances in artificial intelligence and predictive models may present alternative resource light methods of margin personalisation.

Other than patient-specific variables, prolonged oART workflows are a significant barrier to reducing PTV margins in bladder radiotherapy. The target coverage achieved in this study is limited to the workflow speed. Median intra-fractional bladder volume increase has been quantified at 0.8 cc/min in an average workflow time of 19.1 min in CTgART [17], consistent with this study's evaluation, which reports 0.5 cc/min in a median workflow time of 25.6 min in MRIgART. While reported median rates of bladder filling could be considered to be of low significance, other intra-fractional anatomical changes as in patient 4 (Fig. 2) demonstrate that an increase in efficiency could benefit target coverage. Future analysis should consider correlating workflow times with coverage outcomes.

Across both oART platforms, persistent workflow bottlenecks include online contouring, plan optimisation, and software constraints. Workflow times can be further increased by variables such as patient needs, complexity of disease, technical malfunction, which, without accurate methods of verification imaging or gating, could compromise target coverage. Success in efficiency relies on implementing high-quality AI-driven workflows, including auto-segmentation and auto-planning [24]. Such technological advancements are not yet readily integrated into MRIgART systems, these innovations remain the responsibility of oART vendors to reduce the implementation burden on healthcare services. This is not only essential for margin reduction, but also necessary to increase patient access to oART in the context of patient throughput and the rising incidence of cancer.

The ultimate solution to intra-fractional filling is real-time adaptive radiotherapy [25]. While commercial MR-linacs typically offer treatment gating and opportunity for intra-fractional plan shift corrections, effectively accounting for rigidly moving targets during treatment, the adaptations cannot currently account for target deformation once beam delivery is initiated. Future developments in real-time oART will likely further reduce normal tissue exposure, potentially negating the need for margin personalisation and offering an opportunity for further, safer margin reduction.

Limitations of this study are acknowledged, including its sample size, the imbalance in fractions analysed between the 20fr and 6fr cohorts, and the single-centre setting. Further evaluation should include a larger number of patients to draw more robust conclusions, given the significant variability in factors affecting bladder deformation from patient to patient. The edited CTV structures are also subject to risk of inter-observer variability, as edited by one RTT. All CTV structures that failed to meet mandatory constraints were further reviewed by an RO with no or minor feedback. As noted, for 19 fractions MR_POST_ images were either not acquired because an alternative sequence was required to be taken for other analysis (*n* = 3), treatment termination due to machine malfunction (*n* = 2) or patient tolerance (*n* = 2). Erroneously not acquired (*n* = 11) or acquired and unusable due to image distortion (*n* = 1). The potential bias that may have arisen because of missing images is acknowledged.

The feasibility of 5 mm PTV margins is specific to whole bladder radiotherapy; further analysis should be undertaken for stereotactic radiotherapy. Equally, this analysis may not apply to bladder tumour-focused treatment delivery, which often utilises a full bladder protocol to optimise tumour visualisation, as various studies have reported increased intra-fractional filling versus empty bladder protocols [3], [17].

While this study demonstrates feasibility, the safety of margin reduction studies is pivotal. Our study is a dosimetric analysis of target coverage only. Previous studies have demonstrated the benefit anticipated to normal tissue irradiation when PTV margin reduction is employed for bladder radiotherapy [1], [2], [5], [8]. Ongoing patient recruitment to trials in progress will ensure that clinical impact both in terms of disease control and side effects will be reported (Prospective Evaluation of Radiotherapy Using Magnetic Resonance Image Guided Treatment, PERMIT; NCT03727698, The Multiple Outcome Evaluation of Radiation Therapy Using the MR-Linac Study, MOMENTUM; NCT04075305). This study used clinically implemented PTV margins in POTD adaptive treatments, following a preliminary margin reduction analysis [13]. Verification and intra-fractional cine imaging were also used to ensure appropriate PTV coverage via visual assessment online. Short and long-term clinical data, including quality of life and overall survival, will provide context into the true impact of margin reduction.

## 5 Conclusion

This preliminary data demonstrates that whole bladder MRIgART with 5 mm isotropic PTV margins is technically and dosimetrically feasible in achieving acceptable CTV coverage when selective margin personalisation is made available in this small single-centre cohort. This approach leverages online adaptive radiotherapy to account for patient-specific variability while reducing irradiation of normal tissue in patients with minimal intra-fractional change. Further evaluation will explore patient-specific variables and dose accumulation analysis in a larger patient cohort.

## CRediT authorship contribution statement

**Bethany Williams:** Conceptualization, Methodology, Investigation, Data curation, Writing – original draft, Writing – review & editing, Visualization. **Saleh Sandoughdaran:** Investigation, Writing – review & editing. **Joan Chick:** Writing – review & editing. **Alex Dunlop:** Conceptualization, Methodology, Writing – review & editing. **Trina Herbert:** Writing – review & editing. **Robert Huddart:** Conceptualization, Methodology, Writing – review & editing. **Adam Mitchell:** Conceptualization, Methodology, Writing – review & editing. **Shaista Hafeez:** Conceptualization, Methodology, Writing – review & editing, Supervision. **Helen A McNair:** Conceptualization, Methodology, Writing – review & editing, Supervision, Funding acquisition.

## Ethics declaration

Written informed consent to take part in the study and to publish the article has been obtained from all participants or their legal representatives. The privacy rights of participants have been observed.

This study was performed in compliance with relevant laws, regulatory frameworks and guidelines where the research took place.

Ethics committee approval was not required under relevant laws and institutional guidelines. All patients consented to the use of their images for research under PERMIT (NCT03727698) and MOMENTUM (NCT04075305).

## Declaration of competing interest

This project represents independent research funded by the National Institute for Health Research and Health Education England (HEE/NIHR Senior Clinical Lectureship, Professor Helen McNair, ICA-SCL-2018-04-ST2-002) and supported by the National Institute for Health Research (NIHR) Biomedical Research Centre at The Royal Marsden NHS Foundation Trust and the Institute of Cancer Research, London. The views expressed are those of the author(s) and not necessarily those of the NHS, the National Institute for Health Research or the Department of Health and Social Care.

The Royal Marsden Hospital and The Institute of Cancer Research are members of the Elekta MR-Linac Consortium, which aims to coordinate international collaborative research relating to the Elekta Unity (MR- Linac). Elekta (Elekta AB, Stockholm, Sweden) and Philips (Philips, Best, the Netherlands) are commercial members of the MR-Linac Consortium. Elekta financially supports consortium member institutions with research funding, education and travel costs for consortium meetings.

Dr. S Hafeez is the bladder tumour site group chair within the MR-Linac consortium. No commercial financial support was received from any organisation for the submitted work.

Professor R. Huddart reports CRUK, Roche, and MSD funding for bladder RT trials.

Alex Dunlop is a NIHR Senior Clinical and Practitioner Research Award holder.
